# Patient perceptions of the challenges of recruitment to a renal randomised trial registry: a pilot questionnaire-based study

**DOI:** 10.1186/s13063-021-05526-9

**Published:** 2021-09-06

**Authors:** Ellen Murphy, Aoife O’Keeffe, Niamh O Shea, Eva Long, Joseph A. Eustace, Frances Shiely

**Affiliations:** 1 TRAMS (Trials Research and Methodologies Unit), HRB Clinical Research Facility, University College Cork, Cork, Ireland; 2 Department of Nephrology, Cork University Hospital, Cork, Ireland; 3 HRB Clinical Research Facility and School of Public Health, University College Cork, 4th Floor Western Gateway Building, Western Road, Cork, Ireland

**Keywords:** Trial methodology, Registry-based randomised controlled trials, Recruitment, Renal dialysis

## Abstract

**Background:**

Randomised controlled trials (RCTs) are the gold standard for demonstrating the efficacy of new therapies. However, issues of external validity often affect result application to real-world settings. Using registries to conduct RCTs is a reasonably new practice, but is appealing because it combines the benefits of both observational studies and RCTs. There is limited literature on patient motivators, barriers, and consent to registries for conducting RCTs. The purpose of our study was to establish the factors that motivate and/or inhibit patients from joining a registry for RCTs and to determine what information matters to patients when making an enrolment decision to participate in such a registry.

**Methods:**

We conducted a cross-sectional questionnaire-based study at a dialysis centre in Southwest Ireland representing a catchment patient population of approximately 430,000. Quantitative data were coded and analysed in SPSS (v16). Descriptive statistics were produced, and open-ended questions were analysed by thematic analysis.

**Results:**

Eighty-seven patients completed the questionnaire. Reasons for participation in a registry included personal and altruistic benefits. Barriers to participation were time and travel requirements associated with registry participation, data safety concerns, risks, side effects, and concerns that registry participation would impact current treatment. Although 29.8% of patients expressed concern regarding their data being stored in a registry, 79.3% were still willing to consent to have their data uploaded and stored in a registry for conducting RCTs. It was important to patients to have their GP (general practitioner) involved in the decision to participate, despite little day-to-day contact with their GP for renal dialysis management.

**Conclusion:**

Challenges to recruitment to registries for RCTs exist, but addressing the identified concerns of potential participants may aid patients in making a more informed enrolment decision and may improve recruitment to registries, and by extension, to RCTs conducted using the registry.

**Supplementary Information:**

The online version contains supplementary material available at 10.1186/s13063-021-05526-9.

## Background

Randomised controlled trials (RCTs) are the gold standard for demonstrating efficacy of new therapies [1]. However, often issues of external validity affect the application of results to real-world situations [2]. Using registries to conduct RCTs is a reasonably new practice [3, 4], attracting attention from trialists and trial methodologists [4–6]. Patient data within registries has the potential to provide an ideal platform for the conduct of RCTs, thus called registry-based randomised controlled trials (rRCTs), due to the availability of case records, participant randomisation, and follow-up data [3, 4, 7, 8]. rRCTs are appealing due to their low cost, significant reduction in trial workload [3, 9, 10], improved generalisability of study findings, ease and rapidity of enrolment, the potential for complete/long-term follow-up [3, 4, 9–16], and the ability to infer causality [12]. Despite their obvious nod towards pragmatism, a recent scoping review found that they are predominantly comparative effectiveness trials [2]. Literature on patient consent to rRCTs [17–19] is limited, especially among patients with end-stage renal disease. Most literature focuses on oncology [18, 20, 21]. There is a need thus to identify barriers and facilitators to enrolling in registries that facilitate RCTs. The PRioRiTy study identified 20 unanswered questions around trial recruitment [22]. The top priority was “How can randomised trials become part of routine care and best utilise current clinical care pathways?” [22]. The objective of our study is to contribute to this evidence base and establish what information matters to patients when making an enrolment decision to participate in a hypothetical registry for conducting RCTs. This will enable appropriate personnel to provide patients with important and relevant information regarding registries for conducting RCTs. This should give patients the confidence to enrol in such registries, and ultimately facilitate the conduct of rRCTs in routine clinical practice.

## Methods

### Questionnaire design

A questionnaire with both closed and open-ended questions was distributed to renal dialysis patients at a major dialysis centre in Southwest Ireland (Additional file 1). The questionnaire was designed by EM, FS, and AOK. Section 1 of the questionnaire evaluated patients’ self-assessed knowledge of terms/concepts associated with RCTs on a 5-point scale (poor, fair, good, very good, excellent). Section 2 asked patients about their views on participating in a kidney randomised trial registry, i.e. a registry for conducting RCTs specifically for patients on renal dialysis, their willingness to discuss and receive information on the registry, methods of receiving this information, questions regarding data storage, likelihood of consenting to a kidney randomised trial registry and why, and concerns about participation. These questions were for a hypothetical registry. Patients were not actually consenting to enrol in a registry. Responses to closed-ended questions were tick box or via a Likert scale. Sufficient space was left to allow patients to respond to open-ended questions.

### Data collection

**Fig. 1:**
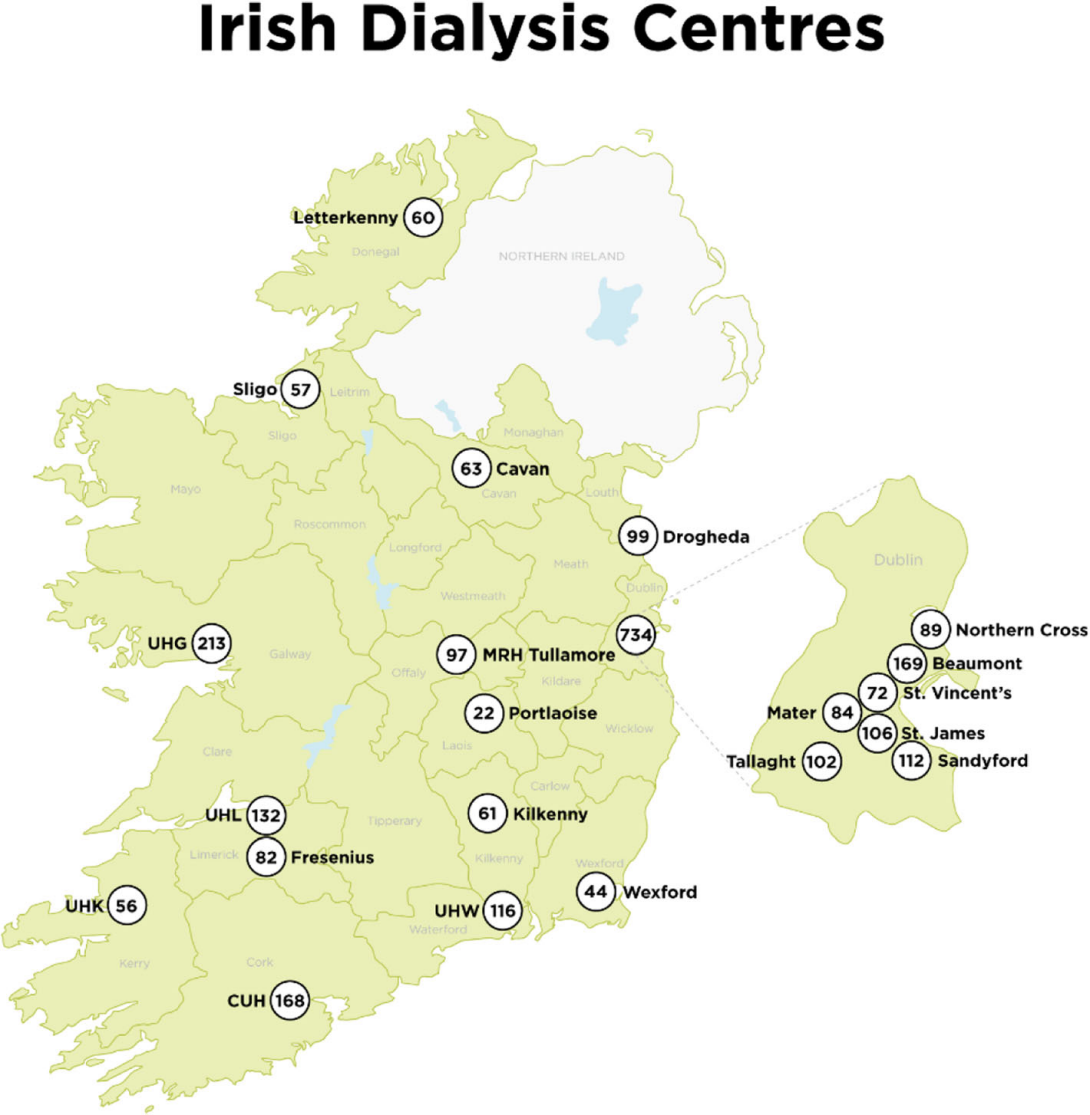
Dialysis centres in Ireland, including numbers of patients dialysed in 2020

The study took place in the CUH (Cork University Hospital) renal dialysis unit in the Southwest of Ireland (Fig. 1). End of year statistics from the National Renal Office show that on 31 December 2020, 2004 patients were receiving haemodialysis in the Republic of Ireland. One hundred and sixty-eight patients, which represents 8.4% of the total haemodialysis population, were dialysed in CUH. Fifty-six patients were dialysed in a satellite unit at UHK (University Hospital Kerry) which is 116 km/72 miles from CUH (Fig. 1). In addition, 18 patients were on home haemodialysis, 30 were on peritoneal dialysis, and 391 were transplant patients. This gave a total of 663 end-stage kidney disease patients under the care of the renal team. Geographical region determines the dialysis site for patients and they attend their closest dialysis unit. In Ireland, the mean distance between patients’ homes and the dialysis unit at which they attend is 29 km [23]. Thus, we have no reason to believe our study sample is not representative of the general dialysis population in Ireland. The CUH dialysis unit opens from 7 am to 12 midnight (3 shifts per day) 7 days a week. Patients in the study were approached and consented to take part in the study in a 6-week period during their normal dialysis schedule (3 times per week; 4-h sessions). Data were collected in the morning, afternoon, and some night shifts, to capture a representative sample of the CUH dialysis population. This is particularly relevant as younger patients with day-time jobs were more likely to be part of the night shift. Dialysis patients are long-term patients and are well known to the nurses in the dialysis unit. Patients that were actively unwell (elderly and frail) and those with cognitive impairment (those that suffered a stroke), as assessed by the senior nurse manager, were not approached to participate. Patients who were sleeping were not disturbed. Eighty-seven patients, 52% of the total CUH dialysis population, completed the questionnaire. Fewer than 5 patients refused to participate, citing feeling unwell/tired as the reason for non-participation.

Patients completed the questionnaire themselves in the presence of a researcher unless they asked for assistance with transcribing answers. In this instance, the researchers (EM and AOK) transcribed the patient’s answers. After completion of section 1 of the questionnaire (answers to Table 1), the researchers provided patients with an explanation of each of the terms: healthcare registry, kidney research registry, clinical trial, randomisation, and informed consent, to ensure clarity for the remainder of the questionnaire. FS compiled the explanations of each term and provided training to EM and AOK on imparting this knowledge to patients. EM and AOK collected the data and used the same explanations to describe the terms to patients. Following these explanations, section 2 of the questionnaire was then completed by the patients.

**Table 1:** Patients’ self-reported understanding of key clinical trial and registry terms/concepts

Research phrase	Self-reported percentage understanding (%)				
	Poor	Fair	Good	Very good	Excellent
**“Healthcare Registry” (*n*= 87)**	18.4%	19.5%	34.5%	20.7%	6.9%
**“Kidney Research Registry” ** **(*n*= 87)**	14.9%	19.5%	37.9%	23.0%	4.6%
**“Clinical Trial” (*n*= 87)**	16.1%	14.9%	32.2%	27.6%	9.2%
**“Randomisation” (*n*= 87)**	34.5%	23%	23%	14.9%	4.6%
**“Informed Consent” (*n*= 87)**	6.9%	10.3%	36.8%	31.0%	14.9%

### Data treatment

Quantitative data were coded and analysed in SPSS (v16). Descriptive statistics were produced. Open-ended questions were analysed by thematic analysis. They were analysed iteratively to explore emergent themes. Thematic analysis [24] was conducted in the first instance by EM with debriefing sessions with co-author FS to discuss similarities or differences in coding labels. This process involved re-reading the transcripts several times which resulted in data immersion [24, 25]. After familiarity, data were coded, then codes were examined for patterns and similarities and grouped together to form themes. The STROBE cross-sectional reporting guidelines were followed to write this manuscript [26].

## Results

### Patient characteristics

All 87 patients were receiving renal dialysis in a hospital setting. Sixty-nine percent were male. The median age was 67 years (range 20–83 years). The mean age was 64 years (SD 14.4). This patient population is broadly representative of the wider population eligible for participation in the registry. A similar study conducted in another Irish hospital in 2019 had a similar mean age, 65 years [27].

### Quantitative findings

#### Patient understanding of trial and registry-related terminology

Patients were asked to assess their understanding of terms/concepts related to RCTs (Table 1). The term registry, whether a “healthcare registry” or a “kidney research registry”, was not well understood. 37.9% and 34.4% of patients reported a “poor/fair” understanding of “healthcare registry” and “kidney research registry” respectively, with only 27% having a “very good/excellent” understanding of each term. Over a third (36.8%) of patients had a “very good/excellent” understanding of “clinical trials”. “Randomisation” was poorly understood with 57.5% of patients reporting only “poor/fair” understanding. Patients showed greatest understanding of “informed consent” with more than 80% having a “good/very good/excellent” understanding of its meaning.

### Patient’s openness to receiving information on a kidney randomised trial registry, from whom, and how

91.7% of patients “strongly agree/agree” to receive and 89.5% “strongly agree/agree” to discuss information about potential participation in a kidney randomised trial registry during dialysis/during a regular clinic visit. Less than 3% “disagree” and 7% were ambivalent. Seventy-six percent of patients “strongly agree/agree” to being contacted by telephone outside of working hours by a researcher to discuss participating in a kidney randomised trial registry. Eighty-six percent of patients “strongly agree/agree” to receiving information by post with an option to discuss it at the next dialysis/clinic visit.

**Fig. 2:**
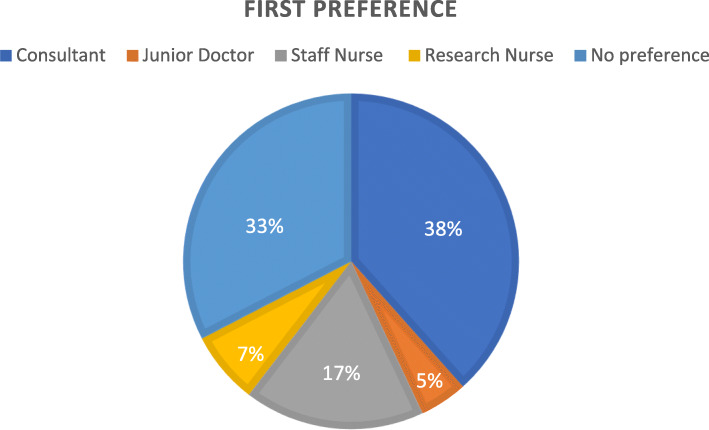
Patients’ communicator preferences for receiving/discussing information about kidney randomised trial registry

54.1% of patient’s preferred method of receiving information about the kidney randomised trial registry was receiving verbal information during their dialysis treatment with the option to consent to the kidney randomised trial registry after the discussion. 28.2% of patients preferred to receive information by post (consent at next visit to the dialysis unit). The least popular methods to receive information were by email (9.4%) and by telephone (8.2%). Figure 2 represents patients’ preferences from whom they would like to receive and discuss information with about the kidney randomised trial registry. Consultants represent the largest group of professionals with whom patients would prefer to receive/discuss information about potential registry participation with.

### Patient data storage concerns

29.8% of patients “strongly agree/agree” to having concerns about their medical data being stored in a kidney randomised trial registry. As a result, 24.1% of patients “strongly agree/agree” to not wanting their data uploaded and stored in a kidney randomised trial registry as they considered their information private. Despite that, only 10.3% of patients were “not likely/very unlikely” to consent to their medical information being uploaded and stored in a kidney randomised trial registry (Table 2).

**Table 2:** Patients’ views on their data being uploaded and stored in a kidney randomised trial registry

	Strongly disagree (%)	Disagree (%)	Neither agree nor disagree (%)	Agree (%)	Strongly agree (%)
**I would have concerns about my medical data being stored in a kidney randomised trial registry (*n*= 87)**	11.5	44.8	13.8	26.4	3.4
**My medical information is private and I do not want it uploaded to a kidney randomised trial registry (*n*= 87)**	12.6	46	17.2	21.8	2.3
	**Very unlikely (%)**	**Not likely (%)**	**Neutral (%)**	**Likely (%)**	**Very likely (%)**
**How likely would you be to consent to your medical information being uploaded and stored in a kidney randomised trial registry (*n*= 87)**	5.7	4.6	10.3	56.3	23

### Patient perspectives on participation and healthcare/carer’s influence

In section 2 of the questionnaire, patients read a short paragraph that explained the consent process, the benefits of signing up to a kidney randomised trial registry, and randomisation, in more detail (see Additional file 1). Researchers EM and AOK assisted with the explanations. Following this, 37.9% of patients were “very likely”, 42.5% were “likely”, 13.8% were “neutral”, 3.4% were “not likely”, and 2.3% “very unlikely” to join a kidney randomised trial registry.

58.8% of patients thought their dialysis doctors should be involved in conducting clinical trials while 41.2% felt their doctors should focus on patient treatment and let somebody else conduct the trials. When signing up to participate in a kidney randomised trial registry, 67.8% of patients would discuss it with somebody. The majority would discuss it with their spouse/partner (35%), their GP (15%), their child (13.3%), parent (6.7%), or friend (1.7%). 28.3% selected “other” and the top preference was their consultant. 51.2% of patients felt it would be “important/very important” for their GP to be involved in their decision to partake in a kidney randomised trial registry while 48.8% felt it was “of little importance/moderately important”. Regarding patients’ views on getting involved in other aspects of study processes, such as study design or conduct, 62.4% reported it was “important/very important”, while 11.8% said “moderately important” and 25.9% of patients said it was “of little importance”. Finally, 94.7% of patients felt it was “important/very important”, to participate in medical research by means of a clinical trial to improve healthcare treatments for others.

### Thematic analysis

Of the 80.4% of patients who would be “likely/very likely” to consent to participate in a kidney randomised trial registry, 87% provided at least one reason why. Reasons were not ranked.

### Theme: Motivators for participation in a kidney randomised trial registry

#### Self-benefit

Self-benefit was identified as one of the main themes impacting on motivations to participate in the kidney randomised trial registry (*n* = 32). Patients would participate in a kidney randomised trial registry “to help myself”, “beneficial for myself”, “personal benefit”, “own self-interest/benefit”, “to improve my health, “to improve my own situation”, and for “better health”.

Within the theme of self-benefit, patients cited reasons orientated around learning: “to learn more about my condition”, “to understand my condition”, “like to know more about it” (*n* = 1), “to learn better/improve quality of life” (*n* = 4), “for further knowledge”, and for “education”. Patients were also motivated to participate for benefits related to their own care process: “if it would help to get off dialysis”, “reduce dialysis hours”, “so treatments can be given to me correctly”, “knowing you are being looked after by experts in that field”, “to help my own care”, “open to better treatments because I have bad kidneys”, “to improve kidney care”, and “to improve healthcare”.

#### Help research, science, and medical advancement

This was an equally strong theme (*n* = 31). Patients said they would be likely to agree to participate in a kidney randomised trial registry for “research purposes”, because it would be “good for research”, “beneficial for research”, “to help research”, “bettering research”, or making “medical discoveries”. Others said it was because “I love research”, “I believe in research”, it is “good for”, it “helps science”, and it would be “advancing medicine”. Others felt “more research is necessary” and that it is “essential every effort is made to improve the situation for people with kidney issues” or helps to “find a cure for kidney problems” and to “find answers to why they are sick”. Patients also felt it was an “important study”; they would participate because it was “for a good cause…if it is an advantage to the study” and because it is a “valuable research”, it might “find new improved treatments”, and it would be “basically positive to clinical trials (to improve procedures)”.

#### Help others

Helping others was another dominant theme (*n* = 19): “to help others”, “to help someone else”, “to help others on dialysis”, “helping others”, “to improve someone else’s situation”, “to improve others health”, “for others benefit”, “benefit to other patients not in the trial”, or “ultimately be beneficial to all”. Linked to this was the help/benefit of future generations of patients, e.g. “it will help those who come after me”, “to help people in the future”, “so others can benefit in future studies”, and “very important for the future”. Three patients were willing to “help” in general; other patients were “eager to make a contribution”, “excited to participate in something new”, and to “better things”; and another believed that their participation would “save time for staff and patients”. One patient believed participation “would help” as “more information is always good”.

#### Why not do it?

Other patients (*n* = 5) agreed to participate because they believed “there’s no harm in it”, they had “no reason not too”, or they would “be interested” or because if they were “asked to participate”.

### Theme: Concerns of patients when being recruited to a kidney randomised trial registry

#### Risks and side effects

75.9% of patients (*n* = 66) responded. Reasons were not ranked. Risks and side effects were important (11.5%; *n* = 10). Ten patients wanted to know more “about the side effects” and “what level of danger there would be”. Others wanted more trial information (*n* = 8), for example “what is involved in a clinical trial” and “if trials went wrong what would happen”. One patient was concerned about being “a guinea pig in the drug trials”.

#### Registry-related concerns

Concerns about the safety of patient medical information were also cited frequently (*n* = 8): “who would be entitled to view the information”, “how safe is my information”, “what would they do with my information”, and “who has access/who will see the data”. Other registry-related concerns included “if the trial was open to review” and “has this method (of trial recruitment) been used in other areas of research and if so what are the results?”. However, ten patients had “no concerns”, e.g. “doesn’t worry me” and “no questions I have total confidence in my consultant”.

#### Time and commitment

A key piece of information was time and commitment. This was listed by 27.2% (*n* = 18): “how much time would it take”, “how long would it take”, “is there a time requirement”, and commitment—“time and place of meeting”, “where would you have to go”, “when would it be”, and “when would it start and finish”. There were only two responses to why patients would not participate in the kidney randomised trial registry. These two patients had “no interest” and “no complications” and linked with this theme they also cited “distance” and “inconvenience” as reasons not to participate.

#### Benefit for self and others

Personal benefit was also a key piece of information required (22.7%; *n* = 15): “what is the benefit for me” and would it be “beneficial to my kidneys”. Three patients wanted to know “if it will help other patients”, “what benefit would it be for my consultant”, and “how would it help”, and two patients wanted to make sure it would help research if they participated “make sure it is helping research” and “would participation help”.

#### Effect on current treatment

Effect on current treatment was another piece of information patients would seek if being enrolled in a kidney randomised trial registry (15%; *n* = 10): “would my normal treatment be constrained”, patients wanted to know if it would interfere with “my medication”, “my dialysis” or the “times of my dialysis treatment”, and “does it reduce dialysis time”.

## Discussion

This is an important study that explores what information matters to patients when making an enrolment decision to a kidney randomised trial registry. We have shown that patients were willing to take steps to participate in RCTs by consenting to be part of a kidney randomised trial registry. This is vital information for us in Ireland as we move towards embedding dialysis clinical trials in routine clinical care (Priority 1 of the PRioRiTy Study) [22] through the amendment of an existing dialysis patient management system, the Kidney Disease Clinical Patient Management System (KDCPMS), to make it fit for conducting dialysis clinical trials. The key findings of our study are that patients’ understanding of RCT-related concepts is poor and patients want more information on and have concerns about risks, side effects, data safety, and implications for current treatment. Despite these concerns, patients are still likely to participate in a registry for personal and altruistic reasons.

Our findings show that patients’ knowledge and understanding of RCT-associated concepts was poor. Patients had a poor understanding of randomisation, a finding that is well known [28–33]. More than 80% of patients had an “excellent/very good/good” understanding of “informed consent”. This is critical to ensuring that patients’ decision-making is autonomous. This finding contradicts research conducted among patients in other medical areas which show a poor understanding of consent for medical procedures. The finding is also higher than results from a prior meta-analysis, which showed the proportion of patients who understood various components of informed consent ranged from 52.1 to 75.8% [34], though the level of understanding in the meta-analysis is not stated, making direct comparison unreliable. Furthermore, due to the limited number of studies that have been conducted in patients with end-stage renal disease, regarding knowledge and understanding of RCT-associated concepts, our findings should be interpreted in context since the research that we compare our findings to has been conducted among patients in other medical specialties.

Identifying key pieces of information that patients want when getting involved in a registry for conducting RCTs is essential to a successful recruitment. Mitigating any factors that will deter people is also important. Time and travel requirements related to any future RCTs were common concerns/queries in this study. Many patients in the dialysis unit avail of HSE (Health Service Executive (government funded))-provided transport to attend their dialysis sessions. They travel in small groups; therefore, arranging transport to participate in any potential research is an issue. However, we anticipate this finding would not be unique to a dialysis cohort and would apply to all rRCTs for patients requiring ongoing regular treatment, e.g. oncology trials. Trialists need to make time and travel requirements very clear and potentially need to be inventive in the design phase of the study, e.g. multiple data collection on the same day, when considering the trial processes to mitigate this, and to improve recruitment especially of those in rural areas with poor transport links. Additionally, this must be budgeted for.

Barriers and concerns of the patients in this study regarding joining a registry for the purposes of conducting RCTs are mirrored in other literature based on clinical trial participation. Concerns about medical risks, harms, and side effects [35–38]; time requirement and travel commitment concerns [18, 35, 36, 39–41]; and data safety concerns [18, 38] are all noted previously. It was also important to patients to be informed about how registry participation would impact their current care and the treatment they normally receive. These topics are relatively easy to address, and our study suggests that if done well, recruitment should be positive. The question of how to do this well should be investigated separately through SWATs (studies within a trial).

Patients had poor understanding of registries but there is limited data to compare it to. However, it informs future researchers establishing registries of the importance of explaining the overall concept of a disease registry for conducting clinical research. Patients’ understanding of randomisation was particularly poor. This is not new [28–33]. For example, in one trial, only 23% of patients were able to explain what randomisation meant [31]; similarly, only 19.5% of our patients had a “excellent/very good” understanding of randomisation. We feel this lack of understanding is a reason why most patients are willing to discuss (89.5% “strongly agree/agree”) and receive (91.7%) information about the kidney randomised trial registry and associated clinical trials. We have gained a valuable insight here on what to focus on when designing informed consent forms for recruiting patients to a registry for subsequent RCT use.

One-third of patients were concerned about the storage of their medical data, and patients felt it was important to know what would happen to their medical data in the registry. It is reassuring that despite these concerns, 80.4% of patients would still be “very likely/likely” to provide consent to participate in a kidney randomised trial registry. There has been an immense amount of progress in data protection, e.g. GDPR (General Data Protection Regulation) [42] and the new EU (European Union) clinical trials directive which commenced in 2014 [43]. Future research would benefit from further investigation to delve into the nuances of patient’s languishing concerns.

Our findings confirm that patients are often altruistic, which is in line with literature based on clinical trial participation [28, 37, 44–46]. Two of our four emerging themes dealt with helping others or helping science; reasons were also listed by Swedish haemodialysis patients [47]. However, “personal benefit” was also a key motivator. Patients wanted to improve their health and the quality of care they receive, a theme highlighted in previous studies on clinical trial participation [28, 46, 48, 49]. However, there is a paradox here: although patients wanted to take part to benefit themselves and improve their health, they were also concerned about the negative effect that trial participation might have on their health. Trialists must ensure that patients are made aware that participation may not always be beneficial, due to the nature of RCTs, to avoid the issue of therapeutic misconception [46]. This is easily addressed on enrolment to a registry and when providing information to patients. These findings are significant to assist those recruiting to a registry for randomised controlled trials as it facilitates the drafting of the patient information leaflet, ensuring it includes information that is important to patients.

Literature shows that individuals are likely to ask for advice on clinical trial participation from their GP or other physicians before consenting to partake [49]. In addition, healthcare providers’ attitude towards clinical trials has been found to be important to patients when making the consent decision [35, 44]. Our findings concur, with 51% preferring the GP to be involved in their decision to participate in a kidney randomised trial registry. GPs are not involved in the day-to-day care of dialysis patients, and this is likely the reason our finding is slightly lower compared to other literature. For example, in one study, 77% of patients stated they wanted to make the decision to participate in a clinical trial with their doctor and only 14% wanted to make it independently [31]. Trusting the physician is clearly very important to patients, a finding concomitant with the literature [49, 50] and further evidenced by the fact a third of our patients would like to receive information on the registry from their consultant. Targeting healthcare professionals to ensure they have adequate knowledge and information about registries for conducting RCTs and how to relay this to potential participants may be a worthwhile intervention to improve recruitment to these registries. The key thing to note is that it is important to patients that their GP be involved in their decision to enrol.

### Strengths and limitations

The sample size is relatively modest (*n* = 87) consisting of elderly adults (median age 67 years) with underlying health conditions, potentially affecting the external validity and generalisability of the findings. However, the unit’s demographics were broadly representative of the dialysis population nationally and therefore may inform efforts to create a dialysis trial registry that will facilitate rRCTs. Although the sample size was modest, it captured 52% of the available patients. Data were collected in the morning, evening, and some night shifts. This was a strength as younger patients with day-time jobs are more likely to be part of the night shift; therefore, we captured a broad age range (median 67 years; range 20–83 years). To support this claim, a study in 2019 of access-related bloodstream infections in dialysis patients in Ireland had a similar mean age to our sample, 65 years (SD 15 years) compared to 64 years (SD 14.35 years) [27].

We were unable to determine if the patients that refused to participate were demographically or clinically different from the patients that participated, but as the number was small, only a handful of patients (fewer than 5%), we do not believe it affects the generalisability of our findings to all dialysis patients.

Selection bias was a possibility as participation was voluntary: those who participated in the study may have a greater interest in research and be more willing to partake in further research/RCTs compared to those who declined to participate. Some very sick patients filled out the questionnaire with assistance, ensuring representation across disease levels. However, assistance/presence of the researcher may have increased engagement among patients and unintentionally caused response bias, particularly regarding the patients self-rating their understanding of RCT-associated terms. Patients may not have wished to express ignorance in front of the researcher and this may have led to the participant positively answering when reporting their knowledge of trial-associated concepts.

It is hard to be sure that patients truly had a full understanding of trial-related concepts such as “randomisation” and “registry” given their complex nature and the fact we did not objectively measure their knowledge. Rather, the researcher provided explanations as well as providing them in writing as part of the questionnaire. Similarly, we did not validate our questionnaire, and we did not use questions on knowledge of trial concepts that had been previously validated in another study as we were unable to find such a study.

We acknowledge that this small, localised sample has issues of wider representation, and if we were conducting this study again, a national dialysis sample would be beneficial. Online surveys could be considered for use as they would facilitate collecting data from patients across the country. However, due to GDPR, it may prove difficult to get an emailing list of renal dialysis patients, and since only 9.4% of our sample wished to receive information about the registry via email, it possibly indicates a dislike/lack of understanding of technology and that more traditional data collection may be more suited to this target population.

## Conclusion

This study shows that the majority of patients included in this study would be willing to participate in registries for the purpose of conducting randomised controlled trials. According to patients, the most important topics that need to specifically be addressed when establishing a registry for randomised controlled trials are the following: additional time and travel commitments, if participation will impact current treatment, data safety, and the risks and side effects involved. These study findings are relevant and important to stakeholders involved in establishing disease-specific registries for RCTs.

## Supplementary Information

**Additional file 1.** Patient Questionnaire.pdf. Patient questionnaire used to collect data in this study.

## Data Availability

The datasets used and/or analysed during the current study are available from the corresponding author on reasonable request.

## Abbreviations

RCT(s): Randomised controlled trial(s)
ESRD: End-stage renal disease
GP: General practitioner
rRCT(s): Registry-based randomised controlled trial(s)
GDPR: General Data Protection Regulation
EU: European Union
HSE: Health Service Executive
SWATs: Studies within a trial
