# Supplementary material for: Patient perceptions of the challenges of recruitment to a renal randomised trial registry: a pilot questionnaire-based study
Source: Trials. 2021 Sep 6;22:597. doi: 10.1186/s13063-021-05526-9 (PMC8420031; doi:10.1186/s13063-021-05526-9)
Supplement: Supplementary file 1 — Additional file 1. Patient Questionnaire.pdf. Patient questionnaire used to collect data in this study. [file 13063_2021_5526_MOESM1_ESM.pdf]

Questionnaire for Participants

Title of Study: The challenges of recruitment to a randomised trial registry – what information matters to the patient?

Date Questionnaire Completed: DD/MM/YYYY ____________________________

Section 1. Background Information

Age (in years) __________________

Sex: Male ☐ Female ☐

1. **How well would you describe your understanding of the phrase 'Healthcare Registry'**

   Please circle

   Poor Fair Good Very Good Excellent

2. **How well would you describe your understanding of the phrase 'Kidney Research Registry'**

   Please circle

   Poor Fair Good Very Good Excellent

3. **How well would you describe your understanding of the phrase 'Clinical Trial'**

   Please circle

   Poor Fair Good Very Good Excellent

4. **How well would you describe your understanding of the phrase 'Randomisation'**

   Please circle

   Poor Fair Good Very Good Excellent

5. **How well would you describe your understanding of the phrase 'Informed Consent'**

   Please circle

   Poor Fair Good Very Good Excellent

We will now explain each of the above phrases to you. Additionally, we will describe a Randomised Trial Registry. Participants kindly completing this questionnaire will receive the same description of each phrase. The remainder of this questionnaire will seek information relating to participation in a randomised trial registry.

Section 2. Your views on participation in a Randomised Trial Registry

Please circle one for each question:

6. **I would be happy to <u>receive</u> information about being included in a kidney randomised trial registry during my dialysis treatment session at the hospital or my clinic visit.** Please circle

   Strongly Disagree   Disagree   Neither Agree nor Disagree   Agree   Strongly Agree

7. **I would be happy to <u>discuss</u> potentially being part of a kidney randomised trial registry during my dialysis treatment session at the hospital or during my clinic visit.** Please circle

   Strongly Disagree   Disagree   Neither Agree nor Disagree   Agree   Strongly Agree

8. **Who would you rather approach you with information about participation in a kidney randomised trial registry.** Please number in order of preference with number 1 being most preferred:

   Consultant ☐
   Junior Doctor ☐
   Staff Nurse ☐
   Research Nurse ☐
   No preference ☐

9. **Sometimes dialysis occurs out of normal working hours. In that instance, I would be happy to being contacted by telephone by hospital staff with information about potential participation in a kidney randomised trial registry?** Please circle

   Strongly Disagree   Disagree   Neither Agree nor Disagree   Agree   Strongly Agree

10. **I would not object to receiving information about participation in a kidney randomised trial registry via post, with the option to discuss participation at my next visit to the dialysis unit or clinic.** Please circle

    Strongly Disagree   Disagree   Neither Agree nor Disagree   Agree   Strongly Agree

11. **What would be your preferred means of receiving information about being included in a kidney randomised trial registry.** Please tick

    - Oral information during dialysis treatment (with the option to consent after discussion) ☐
    - Information received via post (with the option to consent at next visit to dialysis unit) ☐
    - Telephone contact to discuss participation (with the option to consent at next visit to dialysis unit) ☐
    - Email contact ☐

12. **I would have concerns about my medical data being stored in a kidney randomised trial registry.** Please circle

    Strongly Disagree   Disagree   Neither Agree nor Disagree   Agree   Strongly Agree

13. **My medical information is private and I do not want it uploaded to a kidney randomised trial registry for the purpose of research.** Please circle

    Strongly Disagree   Disagree   Neither Agree nor Disagree   Agree   Strongly Agree

14. **How likely would you be to consent to your medical information being uploaded and stored in a kidney randomised trial registry?** Please circle

    Very Likely   Likely   Neutral   Not Likely   Very Unlikely

15. **How important do you believe participation is in medical research by means of a clinical trial to improve healthcare treatments for others?** Please circle

    Unimportant   Of Little Importance   Moderately Important   Important   Very Important

Please read the statement below and answer the questions that follow which are related to this statement.

A kidney randomised trial registry can facilitate automatic randomisation of patients to randomised clinical trials. In effect, your medical information on the kidney registry (all securely held as per data protection laws) tells us if you are suitable for a trial so it cuts out the pre-screening phase. In a simple kidney clinical trial, you would be randomised to either the intervention or control group, and then you would be approached at this point regarding consent to participate or not. The advantage of this process is that the doctor or nurse taking your informed consent only has to explain the treatment you will potentially receive, thus saving them time. The advantage to you is that rather than explaining all of the treatment options available in the trial (one of which you can only receive in any event) you only hear about the treatment on offer to you, and you make a decision to go ahead with that treatment as part of the trial, or not. When we are asking you about consent to a kidney randomised trial registry, it does not mean that you would be forced to participate if you say yes. It only means that you would be considered suitable, randomised to a treatment group and then you would make the decision to participate or not at that point. Even if you do agree to participate in a kidney clinical trial, or other clinical trial, you <u>always</u> have the right to withdraw from any clinical trial at any point in the study and it does not affect your medical care.

16. **Having read the above statement, how likely would you be to give consent to participation in a kidney randomised trial registry?** Please circle

    Very Likely   Likely   Neutral   Not Likely   Very Unlikely

17. If you answered very likely or likely to the above question, what would be your three main reasons for agreeing to be part of a kidney randomised trial registry?

a. ____

b. ____

c. ____

18. If you answered not likely or very unlikely to question 16, what would be your three main reasons for not agreeing to be part of a kidney randomised trial registry?

a. ____

b. ____

c. ____

19. If I were to approach you and ask you to participate in a kidney randomised trial registry, what are the three key pieces of information which would be of most concern to you that you would like me to discuss with you?

a. ____

b. ____

c. ____

20. Do you think your dialysis doctors should be conducting randomised trials or should they leave that to someone else and get on with your treatment in the hospital? Please tick one option

a. Should conduct trials ☐

b. Should just get on with my treatment ☐

21. If you were considering your participation in a kidney randomised trial registry, is there someone you would choose to discuss this with? Please circle

a. Yes

b. No

22. If yes, who would you discuss it with? Please tick one

a. **Spouse/partner** ☐
b. **Child** ☐
c. **Parent** ☐
d. **GP** ☐
e. **Friend** ☐
f. **Other (please say who)________________** ☐

23. Dialysis patients attend their GP infrequently because most of their care is through the hospital clinics or dialysis units. Do you think it's important for your GP to be involved in your decision to participate in a kidney randomised trial registry? Please circle

Very Important   Important   Moderately Important   Of Little Importance

24. Kidney patients are frequently involved in studies, mostly as the patients in the study, like you are helping me today. I'm interested in finding out if you think it is important that kidney patients should also be involved in other aspects of the study process, such as the design of the study, e.g., if you were to help me formulate the questions for this questionnaire, or the conduct of the e.g., perhaps on an advisory committee overseeing the study processes. A kidney patient in this instance would not have any access to patient data so you wouldn't need to worry about confidentiality. Please circle

Very Important   Important   Moderately Important   Of Little Importance

25. We would like to ask one of your family members to also fill out this questionnaire as we are trying to learn their views on having a family member participate in a kidney randomised trial registry. If you have someone attending with you today, we would also like to give the questionnaire to them. Would you be happy for us to do this? Please circle

a. **Yes (family member name and relationship to patient) ___________________________________**
b. **No**

Do you have any comments?

____

____

____

Thank you for taking the time to complete this questionnaire.
